# Community-Based Implementation of Fit & Strong!: Findings to Inform Statewide Scale-Up

**DOI:** 10.1093/geroni/igaf122.1160

**Published:** 2025-12-31

**Authors:** Julie Bobitt, Andrew DeMott, Susan Hughes

**Affiliations:** University of Illinois at Chicago, Chicago, Illinois, United States; University of Illinois Chicago, Chicago, Illinois, United States; University of Illinois Chicago, Chicago, Illinois, United States

## Abstract

Fit & Strong! (F&S) is an evidence-based group physical activity and health education program designed to improve mobility, strength, and self-efficacy among older adults with arthritis. With ARPA funding from the Illinois Department of Public Health (IDPH), community-based organizations (CBOs) successfully launched the program statewide, engaging over 600 participants. This funding enabled us to undertake a series of qualitative interviews with providers that gave us a an opportunity to gain a deeper understanding of the implementation barriers and facilitators experienced by these CBOs. This project evaluated the acceptability, feasibility, and sustainability of F&S delivery across urban and rural Illinois communities. We conducted semi-structured interviews with staff at 20 organizations that delivered F&S. Interview data were analyzed thematically to identify key implementation factors, including organizational capacity, resource needs, staff training, and participant recruitment challenges. Findings revealed strong enthusiasm for F&S among providers, who cited ways in which F&S could be adapted to meet the needs of different communities and its positive impact on older adult health. Challenges identified included the availability of staffing (exercise instructors are scarce in rural areas), securing sustainable funding, and tailoring the program to meet the needs of rural communities with limited resources. This evaluation provides important insights to inform future strategies for sustaining and expanding F&S delivery in collaboration with community partners. Understanding implementation facilitators and barriers directly from CBOs enables more effective statewide dissemination and is an important building block in the effort to ensure long-term access to evidence-based health promotion programs for older adults.

